# Supplementary material for: DNMTs Are Involved in TGF-β1-Induced Epithelial–Mesenchymal Transitions in Airway Epithelial Cells
Source: Int J Mol Sci. 2022 Mar 10;23(6):3003. doi: 10.3390/ijms23063003 (PMC8951572; doi:10.3390/ijms23063003)

Supporting Information

Supplementary Table S1. Clinical characteristics of subjects (N = 37).

**Characteristics**	**Healthy UP (n = 4)**	**CRSsNP-UP (n = 10)**	**CRSwNP-UP (n = 10)**	**CRSwNP-NP (n = 13)**
**Number of women/men**	3/1	3/7	2/8	4/9
**Age (years, mean ± SD)**	40.3 ± 5.7	46.6 ± 4.2	44.6 ± 6.7	49.3 ± 7.1
**Asthma**	0	0	0	0
**Allergic rhinitis**	0	0	0	0
**Lund–Mackay CT score**	0.75	12.9	16.2	15

UP, uncinate process; NP, nasal polyps; CRSsNP, chronic rhinosinusitis without nasal polyps; CRSwNP, chronic rhinosinusitis with nasal polyps; SD, standard deviation.

Supplementary Table S2. Sequences of PCR primers.

Gene Name		Sequences (quantitative RT–PCR)
DNMT1	Forward	5′-GCT GAA GAT CTG GAT GGG GAA G-3′
	Reverse	5′-GGA CTG ACT CCG GTA ACT GTT C-3′
DNMT3A	Forward	5′-AGT ACG ACG ACG ACG GCT A-3′
	Reverse	5′-CAC GAA AAC GCA CCT CAC CA-3′
DNMT3B	Forward	5′-ACC TCG TGT GGG GAA AGA TCA-3′
	Reverse	3′-AGG TCA CCA AAC CGC TAC C-5′
E-cadherin	Forward	5′-TGC TCT TGC TGT TTC TTC GG-3′
	Reverse	5′-TGC CCC ATT CGT TCA AGT AG-3′
α-SMA	Forward	5′-GGC TCT GGG CTC TGG GCT TCA TC-3′
	Reverse	5′-CTC TTG CTC TGG GCT TCA TC-3′
Fibronectin	Forward	5′-CTT TGG TGC AGC ACA ACT TC-3′
	Reverse	5′-CCT CCT CGA GTC TGA ACC AA-3′
Vimentin	Forward	5′-CTC TTG CTC TGG GCT TCA TC-3′
	Reverse	5′-CTC TTG CTC TGG GCT TCA TC-3′
Snail	Forward	5′-TCT AGG CCC TGG CTG CTA CAA-3′
	Reverse	5′-GCC TGG CAC TGG TAC TTC AC-3′
Slug	Forward	5′-ATG CAT ATT CGG ACC CAC C-3′
	Reverse	5′-AGA TTT GAC CTG TCT GCA GCT C-3′
GAPDH	Forward	5′-GTG GAT ATT GTT GCC ATC AAT GAC C-3′
	Reverse	5′-GCC CCA GCC TTC TTC ATG GTG GT-3′

Supplementary Figure S1.

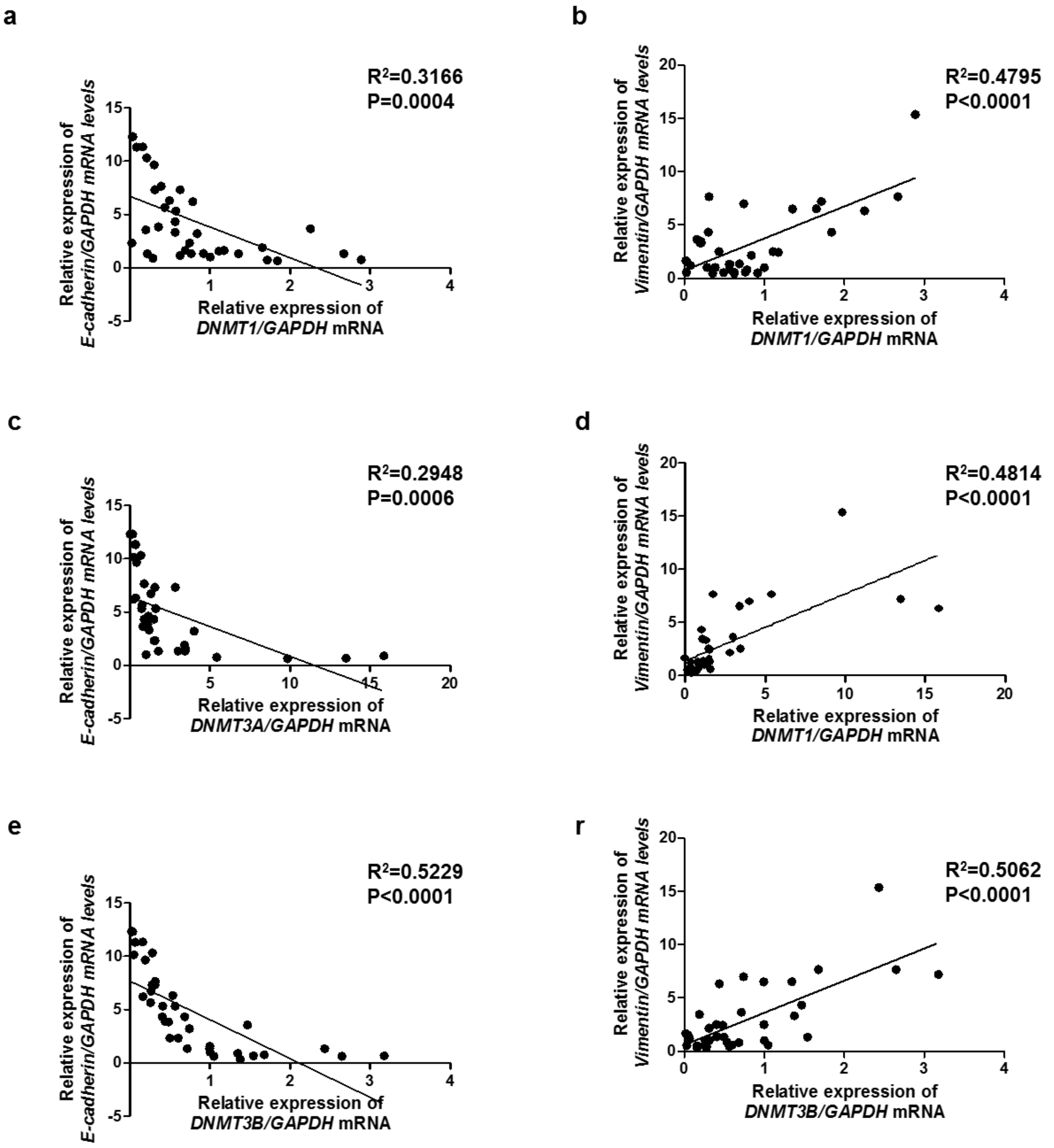

Supplementary Figure S2.

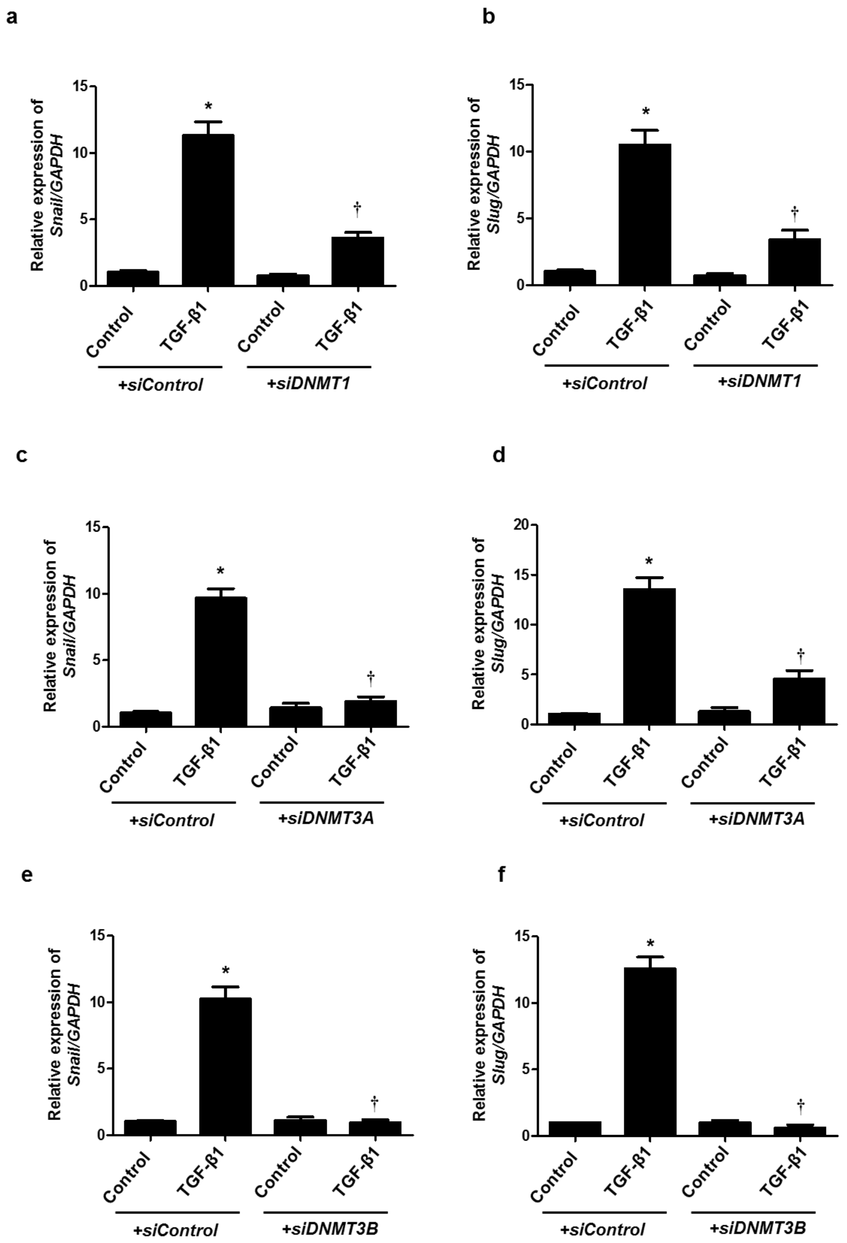

Supplement: Supplementary file 1 [file ijms-23-03003-s001.zip › ijms-1563315-supplementary.pdf]
